# Supplementary figures and images for: A commercial ARHGEF17/TEM4 antibody cross-reacts with Nuclear Mitotic Apparatus protein 1 (NuMA)
Source: PLoS One. 2022 Jul 1;17(7):e0268848. doi: 10.1371/journal.pone.0268848 (PMC9249204; doi:10.1371/journal.pone.0268848)

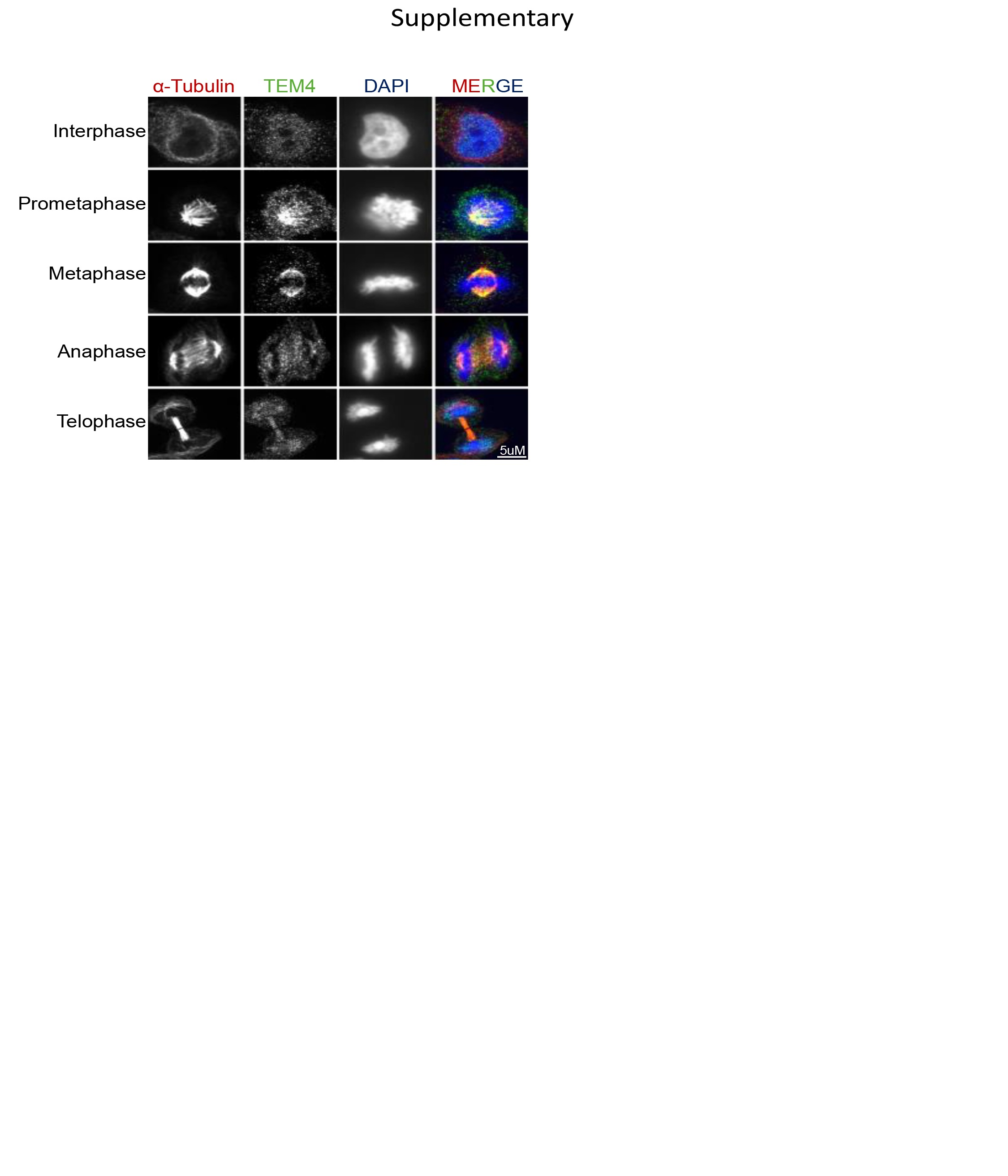

Supplement: S1 Fig — Representative immunofluorescence image of metaphase spindle HeLa-T-REx cell. Cells were synchronized in mitosis after 10 hours of release from double thymidine block and fixed for 10 minutes with PTEMF buffer. Fixed cells were stained with anti-TEM4 (green), anti-α-tubulin for the spindle microtubules (red) and HOECHST to stain the DNA (blue). Scale bar = 5μm. (TIF) [file pone.0268848.s001.tif]

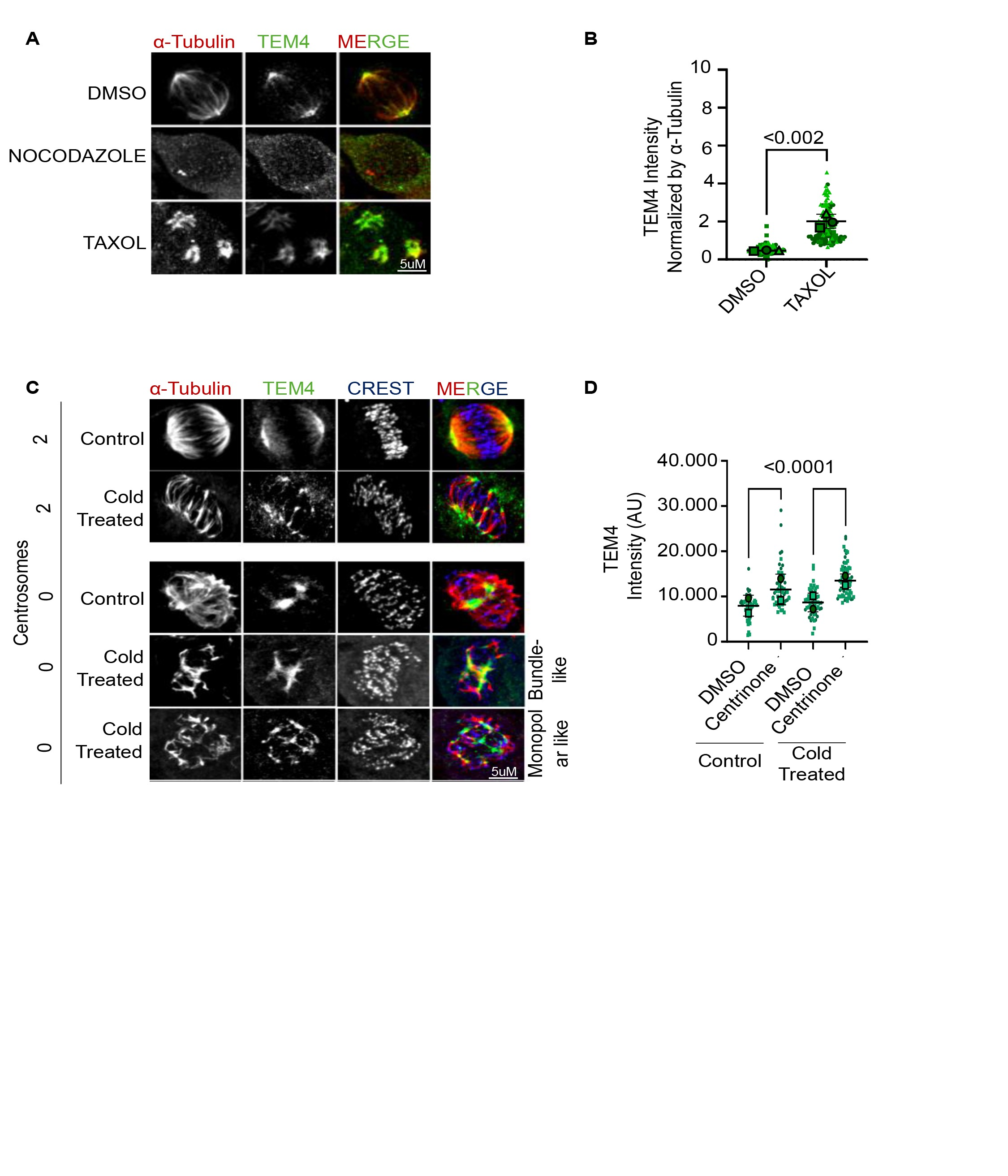

Supplement: S2 Fig — A: Immunofluorescence image of HCT-116 cells treated with 3 μM of Nocodazole or 15 nM of Taxol for 14 hours. Cells were fixed for 10 minutes with PTEMF buffer and stained with anti-TEM4 (green), anti-α-tubulin for the spindle microtubules (red). Scale bar = 5μm. B: Superplot of the quantification of TEM4 intensity at the mitotic spindle in 3 independent experiments corresponding to A. A minimum of 20 cells was counted per condition in each experiment. The P value is calculated using the unpaired t-test. C: Immunofluorescent images of HeLa-T-REx cells treated with DMSO or 100 nM of centrinone for 72 hours. Cells were synchronized in mitosis after 10 hours of release from double thymidine block and 20 minutes before fixation cells were incubated on ice to depolymerize microtubules. Fixation was performed by 10 minutes incubation with PTEMF buffer. Fixed cells were stained with anti-TEM4 (green), anti-α-tubulin for the spindle microtubules (red) and CREST serum for the kinetochores (blue). Scale bar = 5μm. D: Superplot graphs showing quantification of TEM4 intensity at the mitotic spindle corresponding to C. A minimum of 20 cells was counted and two biological replicates were analyzed. (TIF) [file pone.0268848.s002.tif]

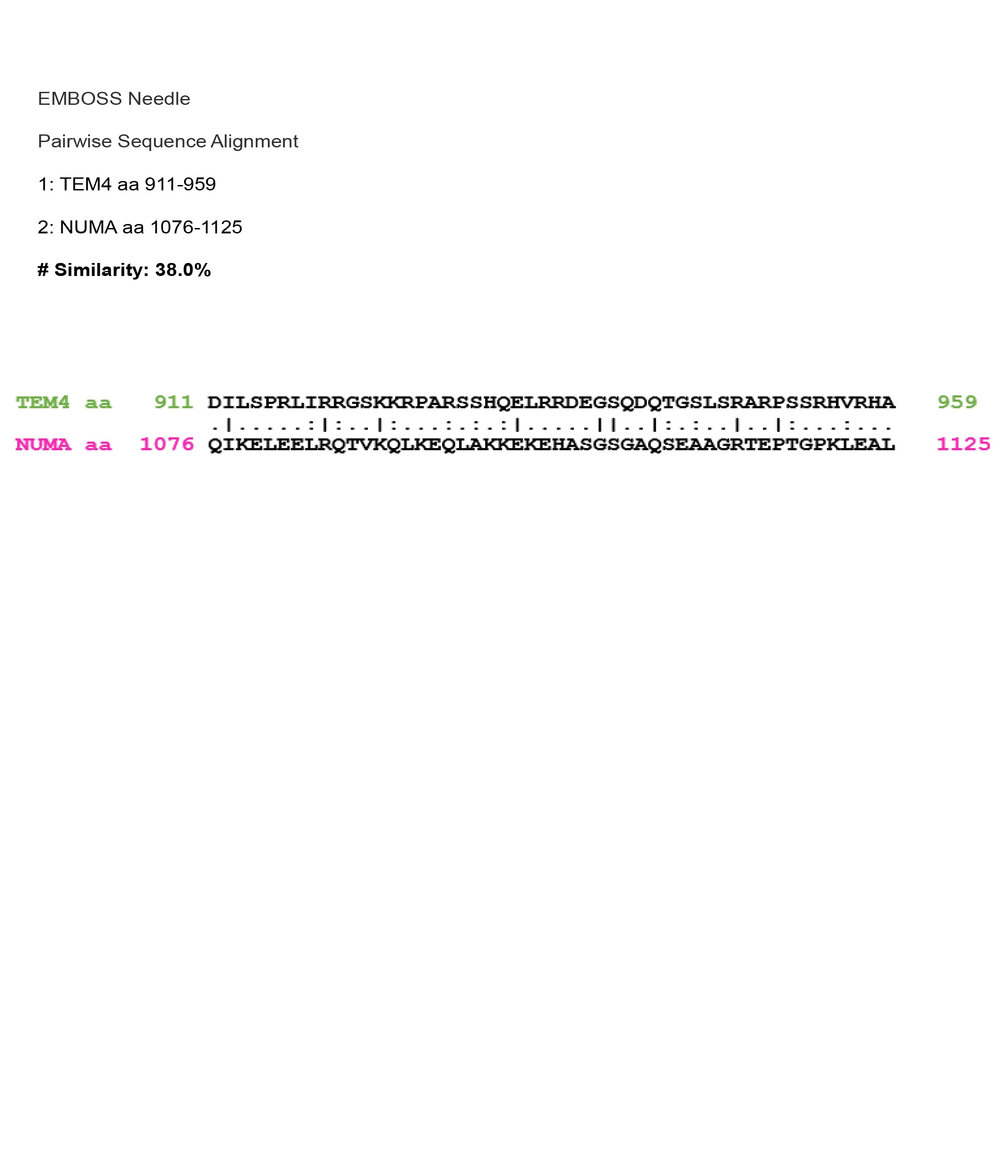

Supplement: S3 Fig — Alignment of TEM4 sequence (residues 911 to 959), (Q96PE2|ARHGH_HUMAN Rho guanine nucleotide exchange factor) and NuMA sequence (residues 1 to 1125), (Q14980|NuMA1_HUMAN Nuclear mitotic apparatus protein 1) using EMBOSS Needle. A pairwise sequence alignment was performed, and the percentage of similarity for those sequences was 38%. (TIF) [file pone.0268848.s003.tif]
