# Supplementary material for: A commercial ARHGEF17/TEM4 antibody cross-reacts with Nuclear Mitotic Apparatus protein 1 (NuMA)
Source: PLoS One. 2022 Jul 1;17(7):e0268848. doi: 10.1371/journal.pone.0268848 (PMC9249204; doi:10.1371/journal.pone.0268848)

All images were captured with ChemiDoc MP Imaging System (Bio-Rad)

Fig. 2 panel C

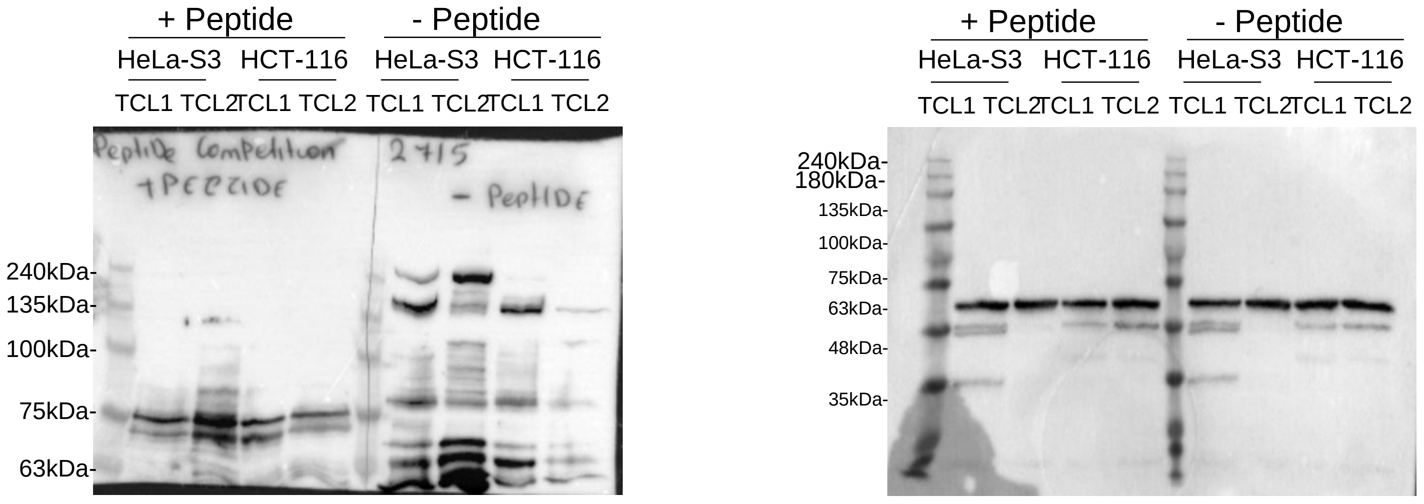

TEM4 α-Tubulin

Fig. 5 panel C

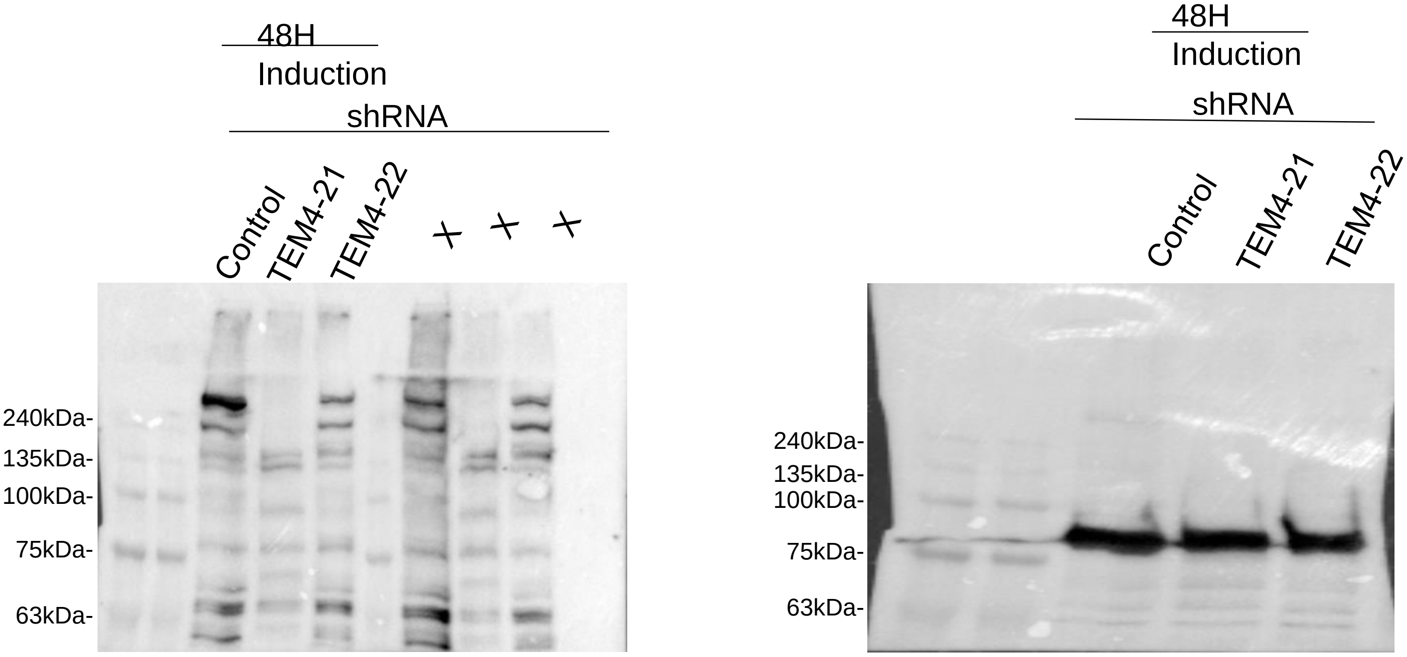

TEM4 Vinculin

Fig. 6 panel B

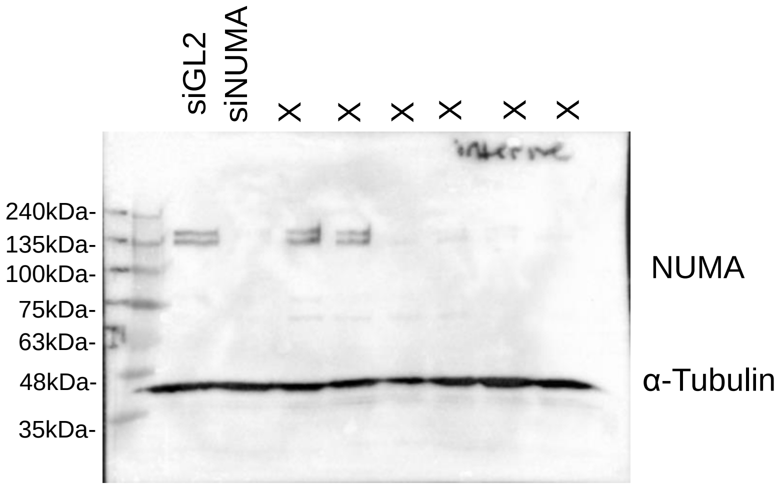

Fig. 6 panel D

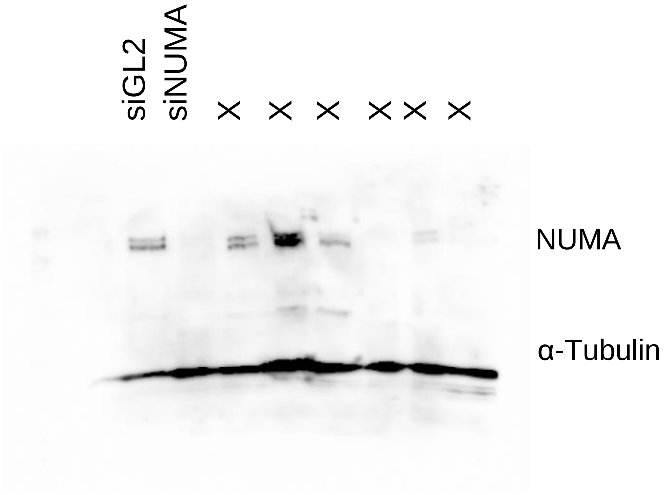

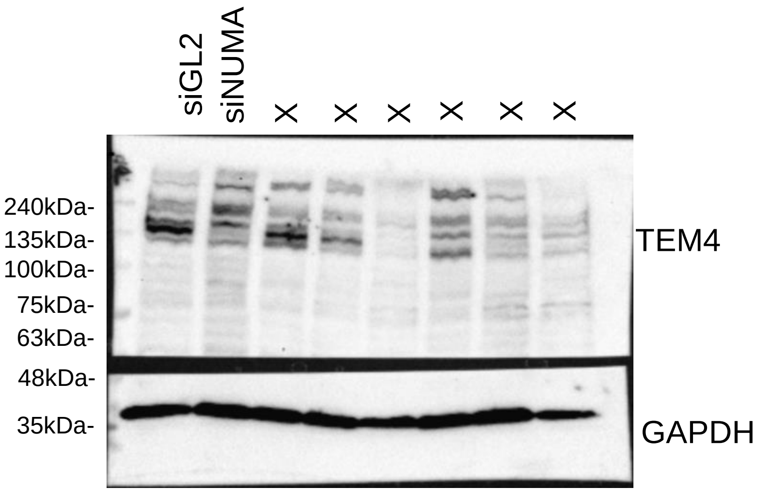

Fig. 7 panel C

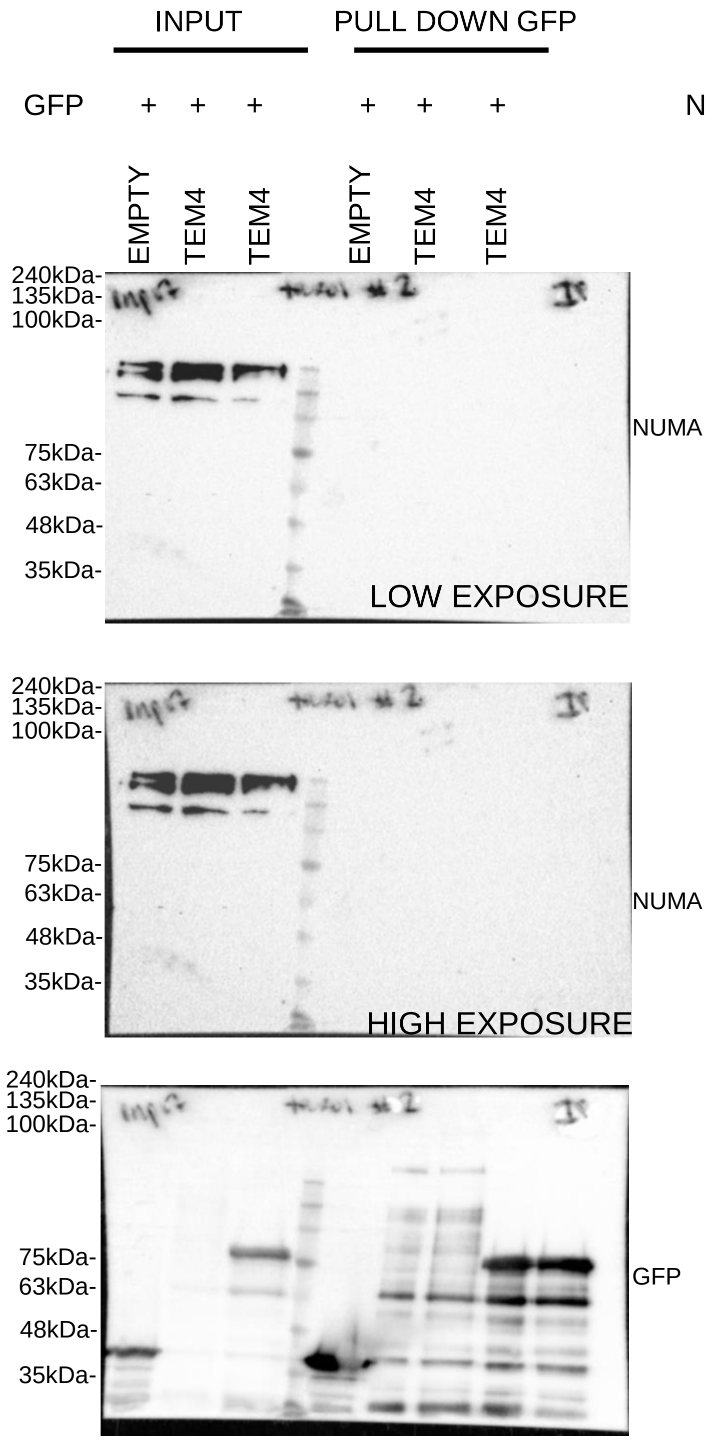

Fig. 7 panel D

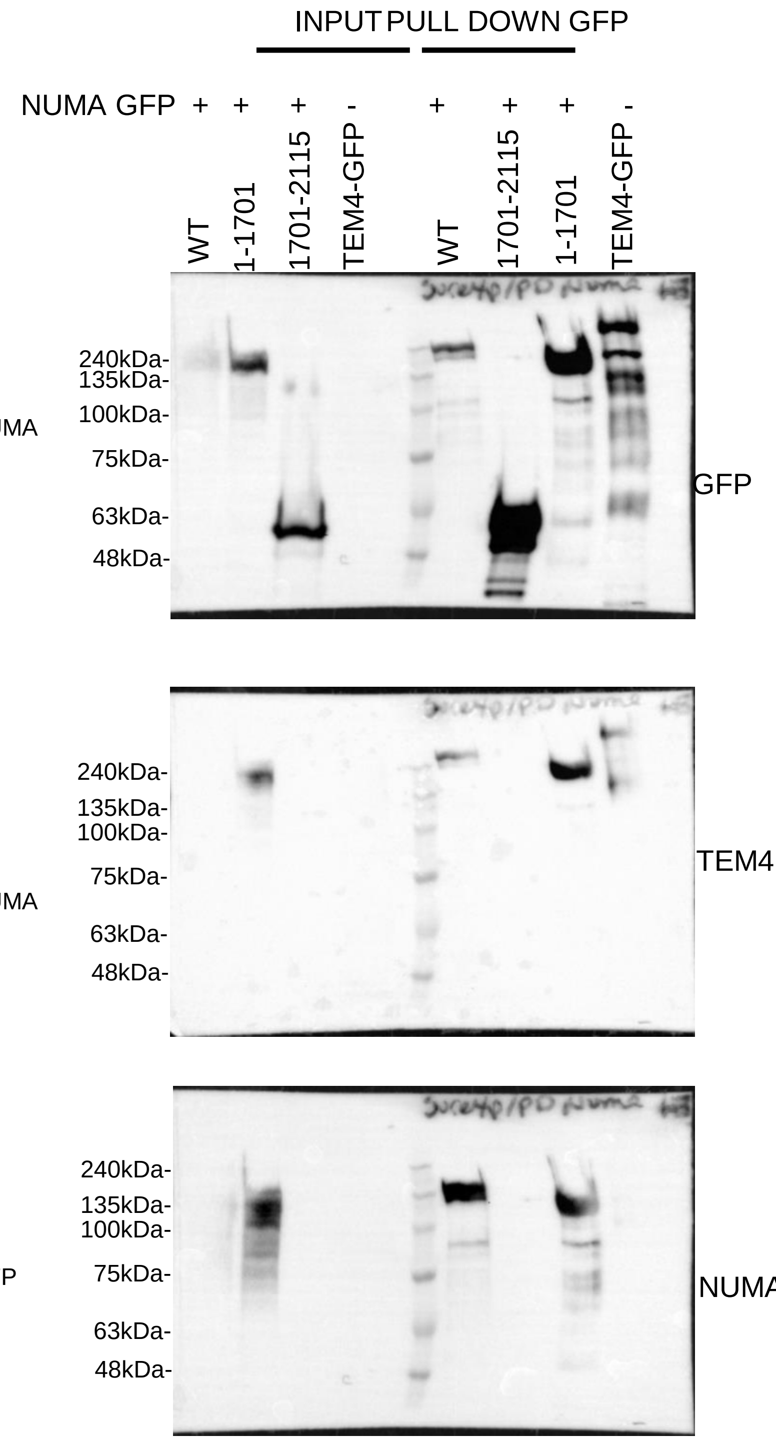

Supplement: S1 Raw images — Unprocessed and uncropped Western blots from Figs 2, 5–7. (PDF) [file pone.0268848.s005.pdf]
