# Supplementary material for: A commercial ARHGEF17/TEM4 antibody cross-reacts with Nuclear Mitotic Apparatus protein 1 (NuMA)
Source: PLoS One. 2022 Jul 1;17(7):e0268848. doi: 10.1371/journal.pone.0268848 (PMC9249204; doi:10.1371/journal.pone.0268848)
Supplement: Peer review history

# Peer review: Decision Letter 0

**DOI:** 10.1371/journal.pone.0268848.r001

**Authors:** Claude Prigent

23 May 2022

A Commercial ARHGEF17/TEM4 antibody cross-reacts with Nuclear Mitotic Apparatus protein 1 (NuMA)

PONE-D-22-13298

Dear Dr. Elowe,

We’re pleased to inform you that your manuscript has been judged scientifically suitable for publication and will be formally accepted for publication once it meets all outstanding technical requirements.

Within one week, you’ll receive an e-mail detailing the required amendments. When these have been addressed, you’ll receive a formal acceptance letter and your manuscript will be scheduled for publication.

An invoice for payment will follow shortly after the formal acceptance. To ensure an efficient process, please log into Editorial Manager at http://www.editorialmanager.com/pone/, click the 'Update My Information' link at the top of the page, and double check that your user information is up-to-date. If you have any billing related questions, please contact our Author Billing department directly at authorbilling@plos.org.

If your institution or institutions have a press office, please notify them about your upcoming paper to help maximize its impact. If they’ll be preparing press materials, please inform our press team as soon as possible -- no later than 48 hours after receiving the formal acceptance. Your manuscript will remain under strict press embargo until 2 pm Eastern Time on the date of publication. For more information, please contact onepress@plos.org.

Kind regards,

Claude Prigent

Academic Editor

PLOS ONE

Additional Editor Comments (optional):

Reviewers' comments:

Reviewer's Responses to Questions

**Comments to the Author**

1. Is the manuscript technically sound, and do the data support the conclusions?

The manuscript must describe a technically sound piece of scientific research with data that supports the conclusions. Experiments must have been conducted rigorously, with appropriate controls, replication, and sample sizes. The conclusions must be drawn appropriately based on the data presented. 

Reviewer #1: Yes

Reviewer #2: Yes

**********

2. Has the statistical analysis been performed appropriately and rigorously? 

Reviewer #1: Yes

Reviewer #2: Yes

**********

3. Have the authors made all data underlying the findings in their manuscript fully available?

The PLOS Data policy requires authors to make all data underlying the findings described in their manuscript fully available without restriction, with rare exception (please refer to the Data Availability Statement in the manuscript PDF file). The data should be provided as part of the manuscript or its supporting information, or deposited to a public repository. For example, in addition to summary statistics, the data points behind means, medians and variance measures should be available. If there are restrictions on publicly sharing data—e.g. participant privacy or use of data from a third party—those must be specified.

Reviewer #1: Yes

Reviewer #2: Yes

**********

4. Is the manuscript presented in an intelligible fashion and written in standard English?

PLOS ONE does not copyedit accepted manuscripts, so the language in submitted articles must be clear, correct, and unambiguous. Any typographical or grammatical errors should be corrected at revision, so please note any specific errors here.

Reviewer #1: Yes

Reviewer #2: Yes

**********

5. Review Comments to the Author

Please use the space provided to explain your answers to the questions above. You may also include additional comments for the author, including concerns about dual publication, research ethics, or publication ethics. (Please upload your review as an attachment if it exceeds 20,000 characters)

Reviewer #1: This study unambiguously shows that the commercial anti-TEM4 antibody (ab67278 from AbCam) cross-reacts with the Nuclear Mitotic Apparatus Protein 1 (NuMA) in western blot, immunoprecipitation and immunocytochemistry. It is essential to alert the scientific community about misleading reagents that could compromise subsequent studies.

Reviewer #2: Somewhat uncharacteristically, I have no major comments on this manuscript. This work convincingly establishes that a widely-used commercial antibody against TEM4 cross-reacts with NuMA. The approach is comprehensive and logical, the experiments are properly controlled, presented data are of high quality. Short of a few typos scattered through the text (e.g., 'Borealin' should be 'Borealis' in line 524), nothing needs to be changed in the manuscript. I really feel for the authors, who clearly were planning to investigate what exactly TEM4 does in mitosis but had to dedicate very significant resources to proving that the standard antibody used in a few previous investigation was unreliable. Reporting this fact is good deed to the entire field and the authors should be commended for their time and effort spent on this "negative" finding.

**********

6. PLOS authors have the option to publish the peer review history of their article (what does this mean?). If published, this will include your full peer review and any attached files.

If you choose “no”, your identity will remain anonymous but your review may still be made public.

**Do you want your identity to be public for this peer review?** For information about this choice, including consent withdrawal, please see our Privacy Policy.

Reviewer #1: No

Reviewer #2: No

# Editor report: Acceptance letter

**DOI:** 10.1371/journal.pone.0268848.r002

**Authors:** Claude Prigent

23 Jun 2022

PONE-D-22-13298 

A Commercial ARHGEF17/TEM4 antibody cross-reacts with Nuclear Mitotic Apparatus protein 1 (NuMA) 

Dear Dr. Elowe:

I'm pleased to inform you that your manuscript has been deemed suitable for publication in PLOS ONE. Congratulations! Your manuscript is now with our production department. 

If your institution or institutions have a press office, please let them know about your upcoming paper now to help maximize its impact. If they'll be preparing press materials, please inform our press team within the next 48 hours. Your manuscript will remain under strict press embargo until 2 pm Eastern Time on the date of publication. For more information please contact onepress@plos.org.

If we can help with anything else, please email us at plosone@plos.org. 

Thank you for submitting your work to PLOS ONE and supporting open access. 

Kind regards, 

PLOS ONE Editorial Office Staff

on behalf of

Dr. Claude Prigent 

Academic Editor

PLOS ONE
